# Magnitude and pattern of facility-based disrespect and abusive treatment of women during childbirth in Abia State, Nigeria

**DOI:** 10.4314/gmj.v56i2.8

**Published:** 2022-06

**Authors:** Kalu U Kalu, Ugochukwu U Onyeonoro, Uche N Nwamoh, Chidinma I Amuzie

**Affiliations:** 1 Department of Community Medicine, Federal Medical Centre, Umuahia, Abia State, Nigeria

**Keywords:** disrespect and abuse, postnatal, healthcare providers, facility delivery, Abia State

## Abstract

**Objectives:**

To ascertain the prevalence and pattern of reported facility-based disrespect and abuse of women during labour in Abia State, South-East, Nigeria.

**Design:**

A cross-sectional questionnaire-based study

**Setting:**

One urban and one rural healthcare facility in Abia State- Federal Medical Centre, Umuahia and Nigerian Christian Hospital (NCH) Nlagu, respectively.

**Participants:**

A total of 312 women who were recently delivered of their babies in the two facilities in Abia State and attending postnatal clinics were sampled for the survey.

**Main Outcome Measure:**

Disrespect and abuse D&A) during labour among women who give birth in healthcare facilities in Abia State.

**Result:**

In all, over half (54.5%) of the women experienced disrespect and abuse in Abia State (50% urban and 63.5% in rural areas). The commonest form of disrespect and abuse was non-confidential care (22.9%). The commonest disrespectful and abusive care received was lack of privacy in the labour ward (18.3%).

**Conclusion:**

The study recorded a high prevalence of reported facility-based disrespect and abuse of women during delivery in Abia State. Training and re-training healthcare providers to promote respectful care, advocacy to policy makers and healthcare stakeholders on the development of respectful maternal care policies and further research in the area are needed.

**Funding:**

No funding was obtained for this study

## Introduction

Respectful maternity care is a critical component of improving maternal health.^1^ Many women, especially those in low-income countries seeking childbirth services, receive various forms of disrespectful and abusive care from their birth attendants, subtle or overt.^2^–^6^ In recent years, there has been growing public attention to the under-reporting of abuse of women during delivery, known as obstetric violence, by many United States (US) health institutions and providers.^7^

Research on the prevalence and nature of disrespect and abuse reveals that disrespect and abuse are a worldwide phenomenon and not just for a selected region.^1^,^4^,^8^–^11^ It is also present across all socio-economic groups, but its prevalence appears to be higher in developing countries, sub-Saharan Africa (SSA) and South Asia.^12^ However, studies on disrespect and abuse during facility-based childbirth are limited. There has been a relative lack of formal research and comprehensive documentation on this topic in Nigeria^13^ and Abia State in particular.

In Nigeria, different forms of disrespect and abuse of pregnant mothers during facility delivery have been documented. These include non-consented services and physical abuse, non-confidential care, detention in the health facility, non-dignified care, abandonment/neglect and discrimination based on some patient's attributes.^14^ Several factors like individual and community beliefs and behaviours, facility sub-systems, provider training and attitudes and the national health system and policies affect the provision of respectful maternity care.^11^ To reduce abuses and improve the care of mothers during childbirth, there is a need to document these practices.^15^ Understanding the burden and patterns of disrespect and abuse will help establish evidence for initiating measures for its reduction. It will also help to identify gaps in the capacity of care providers that need to be addressed to improve care delivery. This will inform the development of appropriate policies, programmes and interventions for its reduction.

Consequently, the study aims to assess the prevalence and pattern of reported facility-based disrespect and abuse experienced by recently delivered women in Abia State, Nigeria.

## Methods

### Study Design and Setting

This cross-sectional study was conducted in two purposively selected urban and rural healthcare facilities in Abia State, Nigeria, the Federal Medical Centre, Umuahia (FMCU) and the Nigerian Christian Hospital (NCH) Nlagu. Federal Medical Centre is an urban settlement tertiary health institution located at the centre of Umuahia town, the capital of Abia state, south-East Nigeria. The facility is a 405-bedded tertiary hospital and one of the leading health care providers in South Eastern Nigeria. The facility is centrally located and readily accessible to people from Enugu, Imo, Cross River, Ebonyi, Rivers, Anambra and Akwa Ibom States. The department's labour ward conducts a monthly average of over 200 deliveries with 122 postnatal visits and has thirteen nurses. The Nigerian Christian Hospital (NCH), a rural secondary-level healthcare centre, is situated on about 119 acres of land at Nlagu, kilometre 18 along Aba-Ikot Ekpene road, Obingwa Local Government Area of Abia State. The Obstetrics and Gynaecology department has one consultant and five senior Medical Officers. The department's labour ward conducts a monthly average of 67 deliveries with 61 postnatal visits and has sixteen nurses.

Both facilities were selected based on their average number of deliveries per month (267). The study population were women of childbearing age whose last delivery was in a health care facility in Abia State, Nigeria. The study was a 3-month survey conducted between March and May 2018.

### Sample Size Calculation

The sample size of 188 per facility was initially calculated from the formula for the prevalence or proportion in a descriptive study for a qualitative outcome variable.^16^ And applying the finite correction formula^17^ for a population less than 10,000, one hundred and forty (140) respondents were recruited for the study. This was necessary because the population for the study was less than 10000. Subsequently, providing for a non-response rate (r) of 10 % (0.1), the sample size was estimated to be156 per facility. Hence, a total of 312 subjects were recruited for both facilities. The subjects were proportionately distributed in the ratio of FMCU:NCH (2:1) based on the delivery records of the respective facilities, thus giving 208 mothers for FMCU and 104 for NCH Nlagu.

### Inclusion/Exclusion criteria

All consenting women still in the puerperium period and attending postnatal care in the selected facilities were recruited. However, mothers who were delivered outside these two facilities were excluded from the study.

### Sampling Technique

The two health facilities were purposively chosen because of their high patient load and high level of utilisation by the majority of the populace in their catchment areas for specialist and general care. They are representative of urban and rural populations. While FMCU serves an urban population, NCH Nlagu serves a predominantly rural population. Using the postnatal clinic register of each facility as the sampling frame, the participants were recruited using a systematic random sampling technique. The sampling fraction was calculated using (N/n) [where n = sample size; N = sampling population)^18^ over three months. Thus, the sample interval was calculated using 3x122/208 and 3x61/104 for FMC, Umuahia and NCH, Nlagu, respectively, thus giving a sampling interval of 2 for both facilities. The average daily clinic attendance was 31 and 16 for FMC Umuahia and NCH Nlagu, respectively, and this formed the daily sampling frame for the selection of subjects. Therefore, at every postnatal clinic session in each facility, the first participant who met the inclusion criteria was selected by simple random sampling (by balloting) from the sampling frame. In contrast, subsequent participants were selected using a systematic random sampling technique where every 2^nd^ woman was approached for selection until the required sample size was attained.

### Data Collection

In addition to the principal researcher, data were collected by a team of six trained research assistants (female nursing students). The research assistants attended a two-day training session before the initiation of the study. The aim of the training, which is part of quality control, was to orient them and ensure a thorough understanding of the study protocol, data collection tools, and informed con-sent procedures. Data were collected using a semi-structured, interviewer-administered questionnaire pre-tested in another urban tertiary health care facility in the State. The questionnaire, which was adapted from a version used in a similar study,^14^ contained mainly definite answers in three sections, including questions on i) Socio-demographics/economic characteristics- age, marital status, tribe, educational status, occupation, religion and average monthly income ii) Obstetric and maternal health service use history and experience during last childbirth;- use of ANC, parity, duration of stay in the facility, history of complication during labour, sex of main HCW during delivery, and number of HCW who attended to the woman during labour and iii) Disrespectful and abusive experiences of the respondents during last delivery. The primary outcome variable was any of the seven (7) forms of disrespect and abuse (irrespective of the type). A woman was considered disrespected and/or abused if she ticked “yes” to any of the questions in the relevant section of the questionnaire. All the questions on the questionnaire relied on the participants' self-report.

### Recruitment Procedure

When the women were seated to receive health talk by the nurses before the postnatal clinic, the introduction of the study personnel took place. The aims and objectives of the study were explained to them. Shortly, the description of why the questionnaire-based approach and how it works was explained to the women. The interviewers then administered the questionnaires to the participants after obtaining their written consent to participate.

### Data Analysis

The data collected was first entered into the Microsoft Office Excel 2015 database, checked for entry errors and then exported to the SPSS statistical software for analysis. Descriptive analysis was done by calculating relevant means and SD for quantitative variables, while categorical variables were expressed in proportions and results presented using appropriate tables.

Ethical approval was sought from the Health Research and Ethics Committee of FMC, Umuahia, with reference number: FMC/QEH/G.596/Vol 10/270. Administrative approval was obtained from the head of NCH, Nlagu. Written informed consent was obtained from each of the participants in the course of the study after due explanation of the survey objective, procedure, risks/benefits and assurance of confidentiality.

## Results

A total of 208 and 104 women from FMCU and NCH Nlagu were recruited for the study. Table 1 shows the socio-demographic characteristic of the study participants. Most of the women were aged between 25–34 years (67.5%) and their mean age was 30.46±5.20 without significant difference in facility location. Two hundred and ninety-five (94.6%) mothers were married; almost all were Christians (99.4%). More than half of the 165 (52.4%) had completed tertiary education, followed by 136 (43.6%) with secondary education. Women in urban areas were more educated than those in rural areas, with 64.4% having tertiary education compared to 29.8% in rural areas. A total of 249 (79.8%) of them were employed, and 205 (65.7%) of them earned at least mini-mum wage ≥ N 18000/month.

**Table 1:** Socio-demographic characteristics of the mothers

Facility Characteristic	FMCU n=208 (%)	NCH Nlagu n=104 (%)	Total n=312 (%)
**Age (in years)**			
≤19	0 (0.0)	4 (3.8)	4 (1.3)
20–24	16 (7.7)	10 (9.6)	26 (8.3)
25–29	85 (40.9)	32 (30.8)	117 (37.5)
30–34	64 (30.8)	30 (28.8)	94 (30.1)
35–39	38 (18.3)	18 (17.3)	56 (17.9)
40+	5 (2.4)	10 (9.6)	15 (4.8)
Mean ±SD	30.3±4.6	30.8±6.2	30.5±5.2
**Marital Status**			
Single	4 (1.9)	7 (6.7)	11 (3.5)
Married	204 (98.1)	91 (87.5)	295 (94.6)
Widowed	0 (0.0)	6 (5.8)	6 (1.9)
**Religion**			
Pentecost	99 (47.6)	52 (50.0)	151 (48.5)
Catholic	76 (36.5)	27 (26.0)	103 (33.0)
Protestant	31 (14.9)	25 (24.0)	56 (17.9)
Islam	2 (1.0)	0 (0.0)	2 (0.6)
**Highest Educational Status Attained**			
None	0 (0.0)	1 (1.0)	1 (0.3)
Primary	3 (1.4)	7 (6.7)	10 (3.2)
Secondary	71 (34.1)	65 (62.5)	136 (43.6)
Tertiary	134 (64.4)	31 (29.8)	165 (52.9)
**Occupation**			
Employed	166 (79.8)	83 (79.8)	249 (79.8)
Unemployed	42 (20.2)	21 (20.2)	63 (20.2)
**Income per month**			
None	31 (14.9)	20 (19.2)	51 (16.4)
< N18000	24 (11.5)	32 (30.6)	56 (17.9)
≥ N 18000	153 (73.6)	52 (50.0)	205 (65.7)
**Tribe**			
Ibo	201 (96.6)	93 (89.4)	294 (94.2)
Ibibio	3 (1.4)	9 (8.7)	12 (3.8)
Yoruba	3 (1.4)	2 (1.9)	5 (1.6)
Nupe	1(0.5)	0 (0.0)	1 (0.3)

Table 2 describes the obstetric and maternal health ser-vices use history and experience of mothers during last childbirth. Three hundred and four (97.4%) mothers utilised ANC at the last birth, and most of them were multiparous, and they had an average number of two children. All (99.7%) except one delivered in a health facility during the final delivery and the average duration of stay in the facility after delivery was 3.2±2.5 days, 3.6±3.6 days and 3.3±2.9 days for urban, rural and all, respectively. Complication occurrence was slightly commoner in urban areas (17.3%) than in rural areas (15.4%). Mothers in urban areas were predominantly delivered by male HCW (70.2%), who were mostly doctors, while their rural counterparts were delivered by female HCW (62.5%).

**Table 2:** Obstetrics and Maternal Health Services use history and experience during last childbirth

Facility Characteristic	FMCU n=208 (%)	NCH Nlagu n=104 (%)	Total n=312 (%)
**Use of ANC at last birth**			
Yes	204 (98.1)	100 (96.2)	304 (97.4)
No	4 (1.9)	4 (3.8)	8 (2.6)
**Parity**			
None	63 (30.3)	24 (23.1)	87 (27.9)
1–4	138 (66.3)	66 (63.4)	204 (65.4)
>4	7 (3.4)	14 (13.5)	21 (6.7)
Mean ±SD (Median)	2.3±1.1(2)	2.7±1.5 (3)	2.4±1.3 (2)
**Duration of Stay in a health facility during last birth (days)**			
1–3	149 (71.6)	74 (71.2)	223 (71.5)
4–6	34 (16.3)	12 (11.5)	46 (14.7)
≥7	25 (12.0)	18 (17.3)	43 (13.8)
Mean ±SD (Median)	3.2± 2.5 (2)	3.6±3.6 (2)	3.3±2.9 (2)
**Had Complications during labour**			
Yes	36 (17.3)	16 (15.4)	52 (16.7)
No	172 (82.7)	88 (84.6)	260 (83.3)
**Sex of main birth attendant at last birth**			
Male	146 (70.2)	39 (37.5)	185 (59.3)
Female	62 (29.8)	65 (62.5)	127 (40.7)
**Number of HCW who attended to you at last childbirth**			
2	17 (8.2)	18 (17.3)	35 (11.2)
3	43 (20.7)	33 (31.7)	76 (24.4)
4	47 (22.6)	34 (32.7)	81 (26.0)
5	40 (19.2)	12 (11.5)	52 (16.7)
6	18 (8.7)	3 (2.9)	21 (6.7)
>6	43 (20.7)	4 (3.8)	47 (15.1)
Mean ±SD (Median)	4.8±2.1 (4)	3.6±1.2 (4)	4.4±1.9 (4)

**Figure 1:**
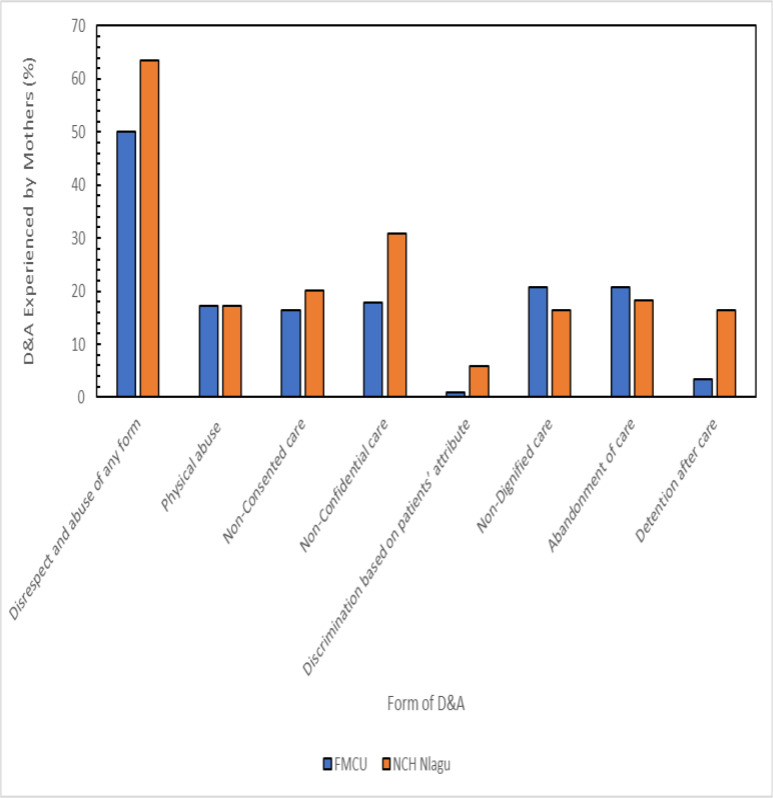
Prevalence and forms of disrespect and abuse experienced by mothers during last childbirth

In general, the prevalence of disrespect and abuse among the women was 54.5% and was higher in NCH Nlagu (63.5%) than in FMCU (50.0%). The commonest form of disrespect and abuse experienced by the mothers during last birth was non-confidential care (22.9%), followed by abandonment of care (19.9%) and non-dignified care (19.2%). In comparison, the least was discrimination based on patients' attributes (2.6%). Prevalence of physical abuse (17.3%) was equal in both facilities; non-dignified care (20.7%) and abandonment of care (20.7%) were more coin mmon in urban areas, and the remainder were commoner in rural areas. Figure 1 shows the prevalence and forms of disrespect and abuse experienced by mothers during the last childbirth

Table 3 shows various specific disrespectful and abusive care experienced by the women during the last childbirth. The commonest form of disrespectful and abusive care experienced by the mothers was lack of privacy in the labour ward (18.3%), denial of companionship (17.0%), threatened with C/S or poor outcome (12.8%), given or sutured episiotomy without anaesthesia (11.9%), scolded or shouted at (11.5%), beaten, slapped or pinched (10.3%) and episiotomy (9.6%) in both facilities. However, while denying companionship was commoner in FMCU, use of episiotomy, lack of privacy in the labour ward, and detention in the facility were commoner in NCH Nlagu.

**Table 3:** Disrespectful and abusive experiences of mothers during last childbirth

Facility Characteristic	FMCU n=208 (%)	NCH Nlagu n=104 (%)	Total n=312 (%)
**Physical Abuse**			
Episiotomy given or sutured without anaesthesia	25 (12.0)	12(11.5)	37(11.9)
Beaten, slapped or pinched	20 (9.6)	12(11.5)	32(10.3)
Tied down or restrained during labour	4 (1.9)	1 (1.0)	5 (1.6)
Sexually abused	1 (0.5)	0 (0.0)	1 (0.3)
**Non-Consented care (services offered without informed consent or** **permission)**			
Episiotomy	15 (7.2)	15(14.4)	30 (9.6)
Augmentation of labour	9 (4.3)	6 (5.8)	15 (4.8)
Shaving of pubic hair	12 (5.8)	4 (3.8)	16 (5.1)
Caesarean Section	8 (3.8)	1 (1.0)	9 (2.9)
Blood transfusion	2 (1.0)	1 (1.0)	3 (1.0)
Sterilisation	2 (1.0)	0 (0.0)	2 (0.6)
**Non-Confidential care (information about woman disclosed to non-** **medics without her permission)**			
No privacy at all in labour ward	29 (13.9)	28(26.9)	57(18.3)
Age	6 (2.9)	6 (5.8)	12 (3.8)
Medical History	6 (2.9)	3 (2.9)	9 (2.9)
Paternity of your child	4 (1.9)	3 (2.9)	7 (2.2)
Ethnicity	1 (0.5)	3 (2.9)	4 (1.3)
HIV Status	2 (1.0)	0 (0.0)	2 (0.6)
**Discrimination on the basis of specific patient attributes**			
Single motherhood status	1 (0.5)	4 (3.8)	5 (1.6)
Low socioeconomic status	0 (0.0)	3 (2.9)	3 (1.0)
Teenage (≤19 years)	2 (1.0)	0 (0.0)	2 (0.6)
HIV Status	0 (0.0)	0 (0.0)	0 (0.0)
**Abandonment/neglect in care**			
Denied companionship in the labour ward	40 (19.2)	13(12.5)	53(17.0)
Not granted requested attention from medics	6 (2.9)	3 (2.9)	9 (2.9)
Left unattended to till second stage of labour	6 (2.9)	0 (0.0)	6 (1.9)
Birth attendant failed to call for help when in danger	1(0.5)	5 (4.8)	6 (1.9)
**Non-Dignified care experiences**			
Threatened with C/S or poor outcome	29 (13.9)	11 (10.6)	40(12.8)
Scolded or shouted at	26 (12.5)	10 (9.6)	36(11.5)
Blamed or intimidated during birth	16 (7.7)	7 (6.7)	23 (7.4)
Received derogatory remarks	16 (7.7)	3 (2.9)	19 (6.1)
**Detention in health facility**			
Detained because could not pay bill	7 (3.4)	15 (14.4)	22 (7.1)
Detained because could not pay bill of child	3 (1.4)	14 (13.5)	17 (5.4)

## Discussion

The study looked at the disrespect and abusive treatment of women by healthcare providers during facility delivery in Abia State. Disrespect and abuse, which is a relative or absolute lack of value for women's lives and health is a known barrier to safe motherhood, as well as a violation of human rights but still remains understud-ied.^19^,^20^ The study showed that the prevalence of disrespect and abuse of pregnant women by healthcare providers during childbirth is 54.5 % in Abia State, with an obvious disparity between the two facilities used in the study — 50% and 63.5% for FMCU (urban) and NCH (rural) facilities respectively.

However, this finding is in contrast with the reported prevalence elsewhere in the sub-Saharan region. The Prevalence of D&A reported in this study is significantly lower than 98% reported among women in Enugu, South Eastern Nigeria,^14^ and 78% reported in Ethiopia.^21^ While our study combined both rural and urban study areas, the other studies were undertaken only in urban dwellings. Also, while the Enugu study was conducted using a convenience sampling method and whose sample size was based on assumption, our study combined utilised scientific sampling methods and sample size calculation.

This probably may have contributed to the huge disparity in the findings and some levels of bias which were sufficiently minimised in our study. Our findings were also at variance with two other studies in Tanzania^22^,^23^ which reported lower prevalence of about 20% and 15%. However, the relatively low prevalence of disrespect and abuse reported in the above studies could be because disrespect and abuse is underreported in rural areas. 14

Our study suggests that reported disrespect and abuse of women during delivery occurs frequently in Abia State and can take the many forms described in the literature for other settings. Of interest is that women can describe and recognise the humiliating experiences they had in the hands of healthcare providers. Non-confidential care was found to be the most commonly reported form of disrespect and abusive treatment by the women in our study, whereas the least frequently reported was discrimination based on patients' attributes. This finding agrees with another study which also reported discrimination based on patients' attributes as the least form of disrespect and abuse because it is regarded as extremes of abuse.^13^

This finding, however, contrasts with a similar study where non-consented care (55%), was the most common specific form of disrespectful or abusive treatment experienced by the women.^14^ ^24^ There is the likelihood that some of these abuses may have been underreported due to acceptance and normalisation of the experience by the women and hence not considered as abuse or disre-spect.^13^,^25^,^26^ Normalisation of these experiences may be attributed to ignorance on the part of these women. On the other hand, the healthcare workers who perpetrate these abuses do so because of a lack of adequate training or re-training. Similarly, it is our belief that the lack of policies on respectful maternity care may have contributed to the high prevalence of D&A in the State in our study. A difference in the pattern of disrespect and abuse was observed between the urban and rural health facilities. While non-dignified care and abandonment in care were more common in the urban health facility, other forms of disrespect and abuse except physical abuse were commoner in the rural area. This finding could be attributed to the low level of education and empowerment of women who reside in rural areas. This group of women usually do not know their rights too.

No mother was discriminated against based on her HIV status both in urban and rural sites. This could be a result of the sustained HIV interventions targeted at reducing of stigmatisation people living with HIV/AIDS (PLWHA) in the area. Besides, both facilities are Prevention of Mother to Child Transmission (PMTCT) facilities; hence their capacities have been built up to sufficiently manage the discrimination and stigma attached to HIV-positive mothers.

It was observed that detention in care for non-settlement of hospital bills is a common practice in both facilities. This could be attributed to a lack of knowledge on respectful maternity care and rights of women by both the healthcare providers and the women and also poor financial power of the women. This situation would be mitigated by instituting free medical services, or at least, a comprehensive health insurance scheme for all pregnant women in the State.

There is a need also to intensify efforts in training and re-training health care providers to discourage them from continuing with the act. Advocacy to policymakers and healthcare stakeholders on the development and implementation of respectful maternity care policies and further research into the factors associated with disrespect and abuse of women during labour is also recommended.

## Limitations and Strengths

The major strength of this study lies in the fact that it is one of the first studies in Abia State to assess the magnitude of disrespect and abuse in the State. However, a few limitations were observed in the study. Interviews were conducted with women whose last delivery was within six (6) weeks and this, therefore, may have introduced recall bias. Responses obtained could not be independently verified as respondents were more likely to report perceived desirable outcomes since they were being interviewed by nurses. Any misunderstanding of the questions in the questionnaire is a potential source of bias inherent with studies relying on self-report. Interviewer bias was reduced by training all the interviewers before carrying out this study.

## Conclusion

The study found a high prevalence of facility-based disrespect and abuse of women during delivery in Abia State. Disrespect and abuse were experienced more in the rural area than in the urban centre. The perpetuation of disrespect and abuse among women during facility delivery over the years has led to its normalisation. Consequently, it is imperative that the stakeholders should design and sustain public health enlightenment programmes on disrespect and abuse (especially among women residing in rural areas) and their fundamental rights to increase their awareness of respectful maternity care.
